# Mucosal immunization with lipopeptides derived from conserved regions of SARS-CoV-2 antigens induce robust cellular and cross-variant humoral immune responses in mice

**DOI:** 10.3389/fimmu.2023.1178523

**Published:** 2023-06-02

**Authors:** Raj S. Patel, Babita Agrawal

**Affiliations:** Department of Surgery, Faculty of Medicine and Dentistry, College of Health Sciences, University of Alberta, Edmonton, AB, Canada

**Keywords:** COVID19, SARS-CoV-2, vaccine, cross-protective, mucosal immunity, cellular immunity

## Abstract

Severe acute respiratory syndrome coronavirus 2 (SARS-CoV-2), the causative agent of COVID-19, has infected >600 million people in the ongoing global pandemic. Several variants of the SARS-CoV-2 have emerged in the last >2 years, challenging the continued efficacy of current COVID vaccines. Therefore, there is a crucial need to investigate a highly cross-protective vaccine effective against variants of SARS-CoV-2. In this study, we examined seven lipopeptides derived from highly conserved, immunodominant epitopes from the S, N, and M proteins of SARS-CoV-2, that are predicted to contain epitopes for clinically protective B cells, helper T cells (TH) and cytotoxic T cells (CTL). Intranasal immunization of mice with most of the lipopeptides led to significantly higher splenocyte proliferation and cytokine production, mucosal and systemic antibody responses, and induction of effector B and T lymphocytes in both lungs and spleen, compared to immunizations with the corresponding peptides without lipid. Immunizations with Spike-derived lipopeptides led to cross-reactive IgG, IgM and IgA responses against Alpha, Beta, Delta, and Omicron Spike proteins as well as neutralizing antibodies. These studies support their potential for development as components of a cross-protective SARS-CoV-2 vaccine.

## Introduction

Severe acute respiratory syndrome coronavirus 2 (SARS-CoV-2), the causative agent of COVID-19, has infected more than 650 million people and caused more than 6.6 million deaths, worldwide (1). The unprecedented spread of the virus spurred the rapid development and implementation of COVID-19 vaccines. The 1^st^ generation COVID-19 vaccines and boosters significantly reduced case numbers, hospitalizations, severe disease outcomes, and mortalities; however, they lacked the ability to prevent SARS-CoV-2 infections (2). Therefore, vaccinated/boosted individuals are still at risk of COVID infections and able to transmit the virus to others. Furthermore, despite the immunity acquired from natural infections of SARS-CoV-2 and vaccines, the virus rapidly mutates to produce highly transmissible and infectious variants, such as Delta, Omicron, and BA.1-BA.5 subvariants, escaping from acquired immunity.

Vaccination is an effective long-term strategy to prevent infections and establish protection against many infectious agents. However, the continuing vaccine development efforts against SARS-CoV-2 appear to be short-sighted, focusing on targeting variants-specific spike (S) glycoprotein as it induces neutralizing antibodies (nAbs). Recent reports suggest that nAb responses, elicited by vaccines and/or natural infections, is an accurate predictor of protection against SARS-CoV-2 (3–5). Generally, as neutralization capacity drops, the risk of fatal outcomes increases, and the absence of early nAbs strongly correlates to mortality and delayed viral control (6). In the long term, nAbs have shown to decline in efficacy due to the emergence of novel SARS-CoV-2 variants with mutations in their spike proteins (7, 8). This has led to periodically updating older vaccines with the latest circulating spike variant and the need for regular booster doses. However, infections by emerging variants cannot be mitigated by limiting vaccine efforts only towards nAbs, that target SARS-CoV-2 S-proteins. Therefore, there is a critical need for a novel broadly-protective vaccine candidate against SARS-CoV-2 and its variants.

Many vaccine studies and reports from recovered COVID-19 patients suggest that cellular immune responses have been associated with protection from disease outcomes and viral control. Immunity from recovered patients reflect that vaccine approaches for SARS-CoV-2 should expand towards targeting conserved proteins such as nucleocapsid (N) and membrane (M) proteins, in addition to the spike protein (9, 10). Furthermore, SARS-CoV-2 specific CD4^+^/CD8^+^ T cells have shown to induce cross-reactive immunity that is resistant to mutations acquired by SARS-CoV-2 variants of concerns (VOCs) and effectively preventing VOC escape (11). For a broadly-protective vaccine, it is vital to investigate novel vaccine candidates, which employ broad cellular and humoral immune mechanisms, and target conserved antigens/regions of SARS-CoV-2 that can be effective against multiple SARS-COV-2 VOCs.

In this study, we identified seven highly conserved, cross-reactive, immunodominant peptides from the S, N, and M proteins of SARS-CoV-2, MERS-CoV, SARS-CoV and other common cold coronaviruses that are predicted to contain epitopes for clinically protective B cells, helper T cells (TH) and cytotoxic T cells (CTL), and which also bind to multiple MHC class I and II molecules (covering >98% of human population) (**Table 1**) (6, 10, 12–27). The lipopeptides corresponding to these peptides containing a lysine-palmitoyl-glycine chain at the carboxy terminus were prepared to enhance immunogenicity. Mono-palmitoylated peptides have been shown to facilitate micelle and antigen depot formation, increase stability and antigen uptake by antigen presenting cells (APCs), and to act as self-adjuvanted molecules that activate PAMP receptors and cross-present antigens on MHC-I and MHC-II (28, 29). Intentionally, selected peptides were 13-15 amino acids in length to facilitate proteolytic processing of antigens and efficient presentation of epitopes by APCs. Here, we compare the immunogenicity of individual SARS-CoV-2 S-, N-, and M-derived lipopeptides with their respective native peptides, using *in vitro* and *in vivo* assays. We found that lipopeptides existed as larger micelle-like particles compared to their peptide counterpart, and stimulated APCs by the upregulation CD40, CD86 and HLA-DR molecules without the activation of TLR-2 and TLR-4 receptors. Intranasal immunizations with individual lipopeptides in mice generated stronger antigen-specific cellular, and mucosal as well as systemic humoral immune responses, compared to their native peptide immunizations. In addition, we provide evidence of the induction of cross-protective immunity upon mucosal immunization with the designed lipopeptides.

**Table 1:** Synthetic lipopeptides and peptides derived from conserved regions of SARS-CoV2 proteins: The viral protein, location, codes, and amino acid sequences.

Pathogen	Protein	Location	Code	Vaccine Construct (Amino Acid Sequence)
SARS-CoV-2	S_1_	492-505	P_1_	LQSYGFQPTNGVGYK(Palmitoyl)G
			P_2_	LQSYGFQPTNGVGY
	S_2_	893-905	P_3_	KRSFIEDLLFNKVK(Palmitoyl)G
			P_4_	KRSFIEDLLFNKV
	N	358-372	P_5_	IDAYKTFPPTEPKKDK(Palmitoyl)G
			P_6_	IDAYKTFPPTEPKKD
		317-331	P_7_	MSRIGMEVTPSGTWLK(Palmitoyl)G
			P_8_	MSRIGMEVTPSGTWL
		158-172	P_9_	VLQLPQGTTLPKGFYK(Palmitoyl)G
			P_10_	VLQLPQGTTLPKGFY
	M	98-112	P_11_	ASFRLFARTRSMWSFK(Palmitoyl)G
			P_12_	ASFRLFARTRSMWSF
		34-48	P_13_	LLQFAYANRNRFLYIK(Palmitoyl)G
			P_14_	LLQFAYANRNRFLYI

## Results

### Lipopeptides self-assemble into sizable, spherical micelle-like particles compared to their native peptides

**Figure 1:**
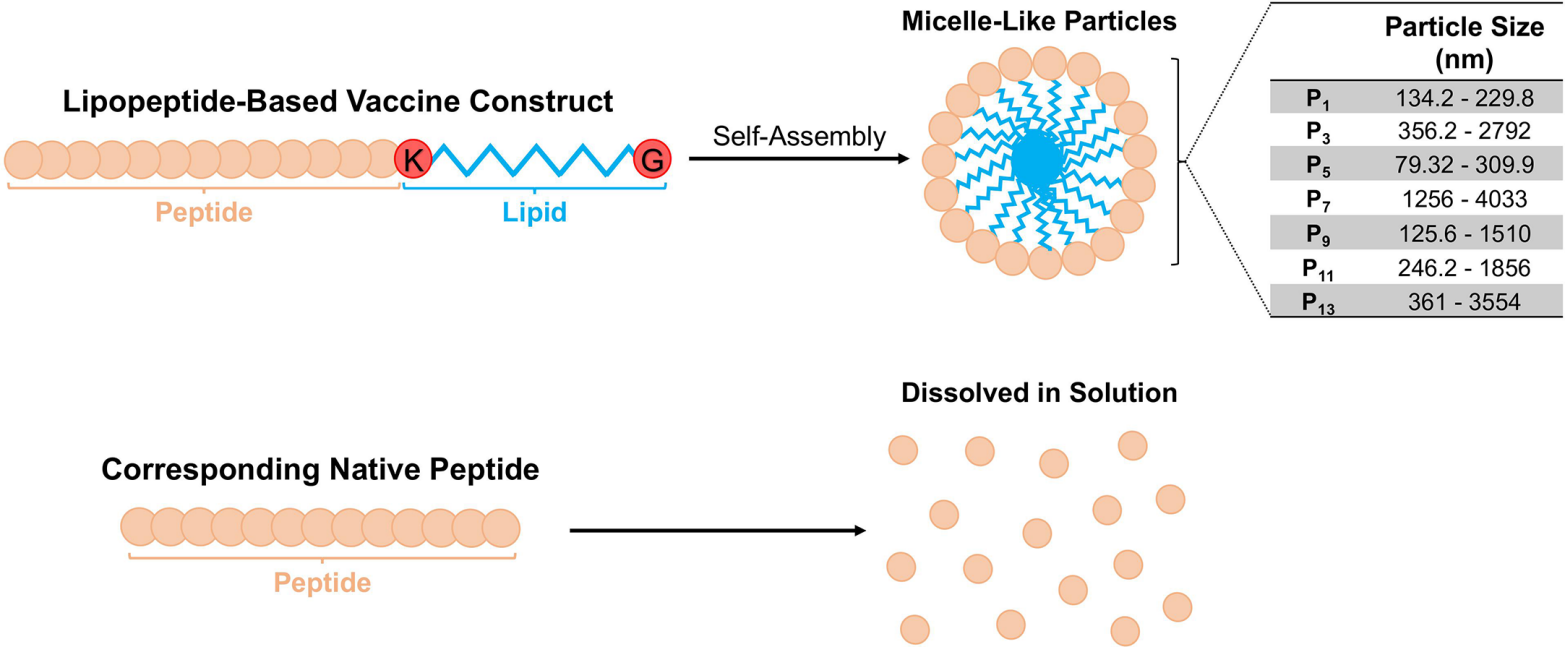
Schematic representation of the lipopeptide-based vaccine construct and particle sizes of P_1_ -P_14_. The physicochemical behavior of the lipopeptide-based vaccine is depicted. The particle sizes of lipopeptides P_1_, P_3_, P_5_, P_7_, P_9_ and P_11_ were determined at different concentrations (0.01, 0.1 and 1.0 mg/ml), and presented as a range from the smallest and largest readings from the tested concentrations.

Based on expected behaviour of palmitoylated lipopeptides in hydrophilic environments, we predicted that the synthetic lipopeptides would aggregate into spherical micelle-like particles (**Figure 1**). Using a ZetaSizer, we examined the size of the lipopeptides’ solution (in PBS) at concentration ranging from 0.01-1.0 mg/ml. At different concentrations, the lipopeptides formed sizable micelle-like particles, whereas the native peptides were completely soluble in PBS. The particle sizes for lipopeptides, P_1_, P_3_, P_5_, P_7_, P_9_, P_11_, and P_13_, ranged from 134.2-229.8 nm, 356.2-2792 nm, 79.32-309.9 nm, 1256- 4033 nm, 125.6- 1510 nm, 246.2-1856 nm, and 361-3554 nm, respectively (**Figure 1**).

### *In vitro* stimulation of antigen-presenting cells with lipopeptides upregulates CD40, CD86, and HLA-DR

**Figure 2:**
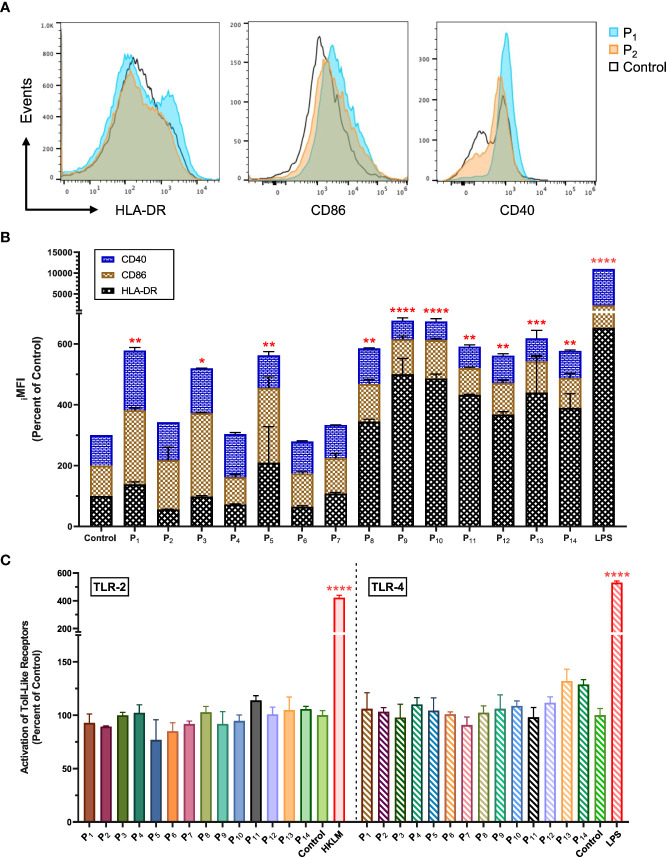
Lipopeptides upregulated CD40, CD86 and HLA-DR on human monocytes without the stimulation of TLR-2 and TLR-4. **(A)** The representative histogram plots of THP-1 cells co-cultured with P_1_(1 μg/ml), P_2_(1 μg/ml), and medium control (RPMI) shows the expression levels of CD40, CD86, and HLA-DR markers. **(B)** The stacked bar graph represents the expression levels of CD40 (blue), CD86 (brown), and HLA-DR (black) for P_1_- P_14_ (1 μg/ml), LPS (1 μg/ml), and control groups (n=3/group). **(C)** Using human TLR-2 or -4 transfected cell lines, the activation of TLR-2 and -4 receptors by P_1_- P_14_ (1 μg/ml) groups (n=3/group) were measured and represented by a bar graph. Positive controls for TLR-2 and TLR-4 were heat-killed *Listeria monocytogenes* (HKLM, 2x10^6^ cfu/ml) and LPS (1 μg/ml), respectively. All data are presented as mean ± SEM of percent of control, from respective experimental control groups. Statistical significance was determined by one-way and two-way ANOVA for **(C, B)**, respectively, followed by a Dunnett’s test (*p ≤ 0.05, **p ≤ 0.01, ***p ≤ 0.001, and ****p ≤ 0.0001 verses Control).

Next, we used flow cytometry to determine whether the lipopeptides could activate APCs (human monocyte cell line, THP-1). We found that P_1_ significantly increased surface expression of CD40, CD86, and HLA-DR on THP-1 cells by 96.6%, 143.0%, and 38.6%, respectively (p=0.0028; **Figures 2A, B**). In comparison, P_2_ increased CD40 and CD86 expression by 25.0% and 61.9%, respectively (**Figure 2B**). LPS stimulated THP-1 cells were used as a positive control group (**Figure 2B**). Other lipopeptides and peptides that increased surface expression of CD40 on THP-1 cells were P_3_ (47.5%), P_4_ (41.7%), P_5_ (7.1%), P_6_ (5.7%), P_7_ (9.6%), and P_8_ (17.3%) (**Figure 2B**). In addition, surface expression of CD86 was increased on THP-1 cells by P_3_ (174.0%), P_5_ (145.5%), P_6_ (9.4%), P_7_ (15.9%), P_8_ (23.5%), P_9_ (13.9%), P_10_ (26.2%), P_12_ (4.1%), and P_13_ (2.4%) (**Figure 2B**). Lastly, surface expression of HLA-DR on THP-1 cells was increased by P_5_ (110.0%), P_7_ (7.9%), P_8_ (244.7%), P_9_ (400.7%), P_10_ (386.9%), P_11_ (332.1%), P_12_ (267.6%), P_13_ (340.0%), and P_14_ (289.7%) (**Figure 2B**). In comparison to controls, there was significant upregulation of CD40, CD86, and HLA-DR surface molecules on THP-1 cells upon lipopeptide stimulation with P_1_ (p=0.0028), P_3_ (p=0.0291), P_5_ (0.0053), P_9_ (p<0.0001), P_11_ (p=0.0015), and P_13_ (p=0.0005), and native peptide stimulation with P_8_ (p=0.002), P_10_ (p<0.0001), P_12_ (p=0.0056), and P_14_ (p=0.0029). THP-1 cells stimulated with lipopeptides, or peptides did not produce detectable levels of proinflammatory cytokines (IFNγ, TNFα, IL-1β etc., data not shown) or stimulate human TLR-2 and -4 receptors (**Figure 2C**). Overall, these results demonstrate that the designed lipopeptides have the capability of stimulating antigen-presenting function without inducing inflammatory cytokine production or TLR stimulation.

### Intranasal immunizations with individual lipopeptides induce robust proliferation and cytokine responses from splenocytes upon *ex vivo* stimulation with their respective peptides

**Figure 3:**
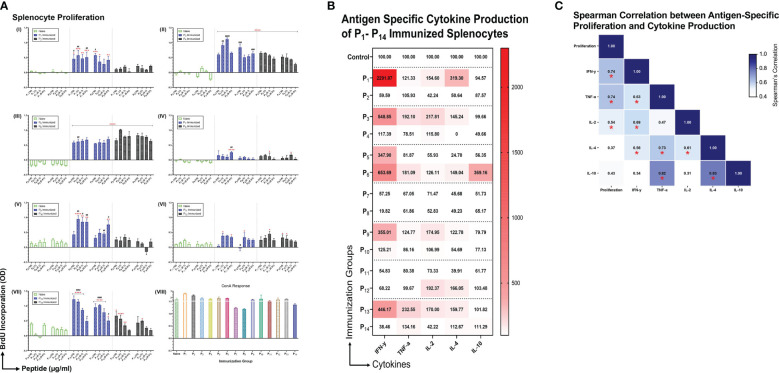
Antigen specific proliferation and cytokine production of P_1_-P_14_ immunized splenocytes. **(A)** Male C57BL/6 mice (n=3/group) were immunized intranasally twice, 14 days apart, with individual lipopeptides and peptides, P_1_-P_14_ (10 μg/mouse) in total volume of 30 μL/mouse. Unimmunized mice were used as controls. Eight days after 2^nd^ immunization, splenocytes were cultured with irradiated APCs (from unimmunized syngeneic mice) and re-stimulated *ex vivo* with its respective lipopeptides and native peptides, at 10, 1, 0.1, and 0.01 μg/ml for 4 days. Proliferation was measured by BrdU incorporation into proliferating splenocytes. ConA (1 μg/ml) was used as a positive control for all groups. Data are shown as mean ± SEM from triplicate wells and represent three independent repeat experiments. Statistical significance was determined by two-way ANOVA, followed by Tukey’s multiple comparison analysis. Comparisons between the immunized and unimmunized groups (*p ≤ 0.05, **p ≤ 0.01, ***p ≤ 0.001, and ****p ≤ 0.0001), and the respective lipopeptide vs. peptide immunized groups (^#^p ≤ 0.05, ^##^p ≤ 0.01, ^###^p ≤ 0.001, and ^####^p ≤ 0.0001) are indicated. **(B)** Culture supernatants from proliferating splenocytes, from individually immunized mice that were re-stimulated *ex vivo* with its respective native peptide at 1 μg/ml were tested for cytokine production (IFN-γ, TNF-α, IL-2, IL-4, IL-10). Supernatants from triplicate cultures were pooled and run-on MSD plates in duplicates. Mean concentrations of duplicates were presented as percent of control values and visualized by heatmaps. **(C)** Spearman’s correlation heatmap shows the association between antigen-specific proliferation from P_1_-P_14_ immunized splenocytes and cytokine production. The Spearman’s correlation coefficient is presented on the correlation matrix, with ‘*’ indicating a significant correlation (p<0.05).

To examine the immunogenicity of the lipopeptides and peptides, mice were immunized intranasally with individual lipopeptides and peptides (10 μg/mouse) twice, 14 days apart. Eight days after the 2^nd^ immunization, spleens were examined for antigen-specific proliferation responses using a colorimetric BrdU incorporation assay. Splenocytes from all immunized groups were also stimulated with ConA (T cell mitogen), which was used as a positive control (**Figure 3A VIII**). Compared to the unimmunized control group, intranasal immunization with P_1_ induced a significantly higher splenocyte proliferation response upon re-stimulation with P_1_ (at 10, 1, 0.1, and 0.01 μg/ml) and P_2_ (at 10, 1, and 0.01 μg/ml) (**Figure 3A I**). In addition, P_1_ immunization had a significantly higher proliferation response compared to its native peptide immunization group, P_2_. Similarly, other intranasal lipopeptide and peptide immunization pairs, including P_3_ and P_4_ (**Figure 3A II**), P_9_ and P_10_ (**Figure 3A V**), and P_13_ and P_14_ (**Figure 3A VII**), demonstrated that splenocytes from lipopeptide-immunized groups have significantly higher proliferative responses upon re-stimulation with corresponding lipopeptides and peptides, compared to the unimmunized controls and corresponding peptide-immunized group. In contrast, P_6_ immunizations induced a slightly higher proliferation response compared to P_5_; while, both immunization groups induced significantly higher proliferation responses, compared to unimmunized controls (**Figure 3A III**). P_11_ and P_12_ immunizations induced a moderate proliferative response, compared to the unimmunized controls, however, the response between the lipopeptide and peptide immunizations were identical (**Figure 3A VI**).

Subsequently, we examined the cytokine profiles of proliferating splenocytes, derived from intranasally immunized mice with P_1_-P_14_, that were restimulated *ex vivo* with their respective native peptides (1 μg/ml). We found that splenocytes from lipopeptide immunizations, P_1_, P_3_, P_9_, and P_13_, produce higher levels of IFN-γ, TNF-α, IL-2, and IL-4, compared to their respective peptide immunization groups (**Figure 3B**). On the contrary, splenocytes derived from P_6_ immunizations induced higher levels of IFN-γ, TNF-α, IL-2, IL-4, and IL-10, compared to P_5_. Lipopeptide immunizations with P_7_ and P_11_ showed no production of cytokines from splenocytes. Moreover, Spearman’s correlation showed levels of IFN-γ (r=0.741, p=0.003), TNF-α (r=0.736, p=0.004), and IL-2 (r=0.538, p=0.05) significantly correlated with antigen-specific splenocyte proliferation responses (**Figure 3C**).

### Intranasal immunizations with individual lipopeptides and peptides elicit mucosal IgA and systemic IgM/IgG antibody response against respective SARS-CoV-2 spike-nucleocapsid fusion, nucleocapsid and membrane proteins

We assessed antibody responses from bronchoalveolar lavages (BALs), and serum samples collected from P_1_-P_14_ immunized mice, eight days after the 2^nd^ immunization. To determine the antigen specificity of the antibody responses, ELISA plates were coated with full length S-N, N, and M proteins of SARS-CoV-2, and BAL or serum samples were tested for IgA, or IgM and IgG antibodies.

**Figure 4:**
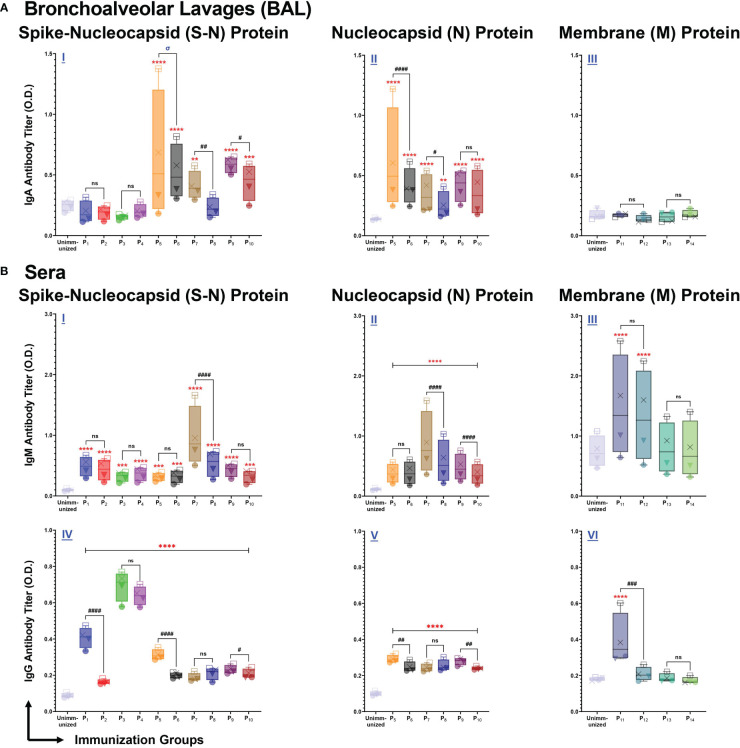
Intranasal immunization with individual lipopeptide and peptides P_1_-P_14_ induce strong antigen-specific humoral responses against the respective spike-nucleocapsid fusion (S-N), nucleocapsid (N), and membrane (M) proteins of SARS-CoV-2. Male C57BL/6 mice (n=3/group) were immunized intranasally twice, 14 days apart. Bronchoalveolar lavages (BALs) and serum samples were collected from groups individually immunized with P_1_-P_14_ eight days after the second immunization. ELISA plates were coated with recombinant antigens: S-N (left), N (middle), and M (right) proteins of SARS-CoV-2 at 1 μg/ml. Plates were incubated with serial dilutions of BALs (□1:2, X1:4, ▼1:8, ●1:16) and sera (□1:100, X1:200, ▼1:400, ●1:800) from each individually immunized group. **(A)** BAL IgA, and **(B)** serum IgM/IgG antibody titers were determined and presented as box and whisker plots. Serum or BAL samples collected from unimmunized mice were used as negative controls. Statistical differences were determined by a two-way ANOVA, followed by a Tukey’s multiple comparison analysis. Comparisons between the immunized and unimmunized groups (**p ≤ 0.01, ***p ≤ 0.001, and ****p ≤ 0.0001), and the respective lipopeptide vs. peptide immunized groups (ns (not significant), ^#^p ≤ 0.05, ^##^p ≤ 0.01, ^###^p ≤ 0.001, and ^####^p ≤ 0.0001) are indicated. ‘σ‘ indicates significance (p ≤ 0.05) only at the two highest dilutions. All data are representative of three independent repeat experiments.

BAL fluid from P_5_ (p<0.0001), P_6_ (p<0.0001), P_7_ (p=0.0041), P_9_ (p<0.0001), and P_10_ (p=0.0004) immunizations elicited a significantly higher anti-(S-N fusion)-IgA antibody response, compared to unimmunized controls (**Figure 4A I**). In addition, we found that lipopeptide immunizations with P_7_ (p=0.0011) and P_9_ (p=0.0242) induced significantly higher anti-(S-N fusion)-IgA antibody titers, compared to their respective peptide immunizations. P_5_ induced a significantly higher anti-(S-N fusion)-IgA titer at a 1:2 dilution, compared to the P_6_ (p<0.0001) (**Figure 4A I**). We found that intranasal immunizations with nucleocapsid-derived lipopeptides and peptides (P_5_-P_10_) led to a significant anti-(N)-IgA antibody titer, compared to unimmunized controls (**Figure 4A II**). Nucleocapsid-derived lipopeptides, P_5_ and P_7_, had significantly higher anti-(N)-IgA titers compared to their respective peptide, with p<0.0001 and p=0.0347, respectively. Nucleocapsid-derived lipopeptide immunizations showed a strong mucosal anti-(S-N fusion)-IgA and anti-(N)-IgA antibody response compared to their respective nucleocapsid-derived peptide immunization. In contrast, membrane-derived lipopeptide and peptide (P_11_-P_14_) immunizations did not elicit anti-(M)-IgA antibodies (**Figure 4A III**).

Next, we examined systemic IgM responses in serum samples collected from P_1_-P_14_ immunized mice (**Figure 4B**). Compared to the unimmunized control group, all spike-derived (P_1_-P_4_) and nucleocapsid-derived (P_5_-P_10_) lipopeptide and peptide immunizations produced significant amounts of anti-(S-N fusion)-IgM antibodies (**Figure 4B I**). In addition, P_5_-P_10_ immunized mice induced significantly higher anti-(N)-IgM antibodies, compared to the unimmunized controls (**Figure 4B II**). Notably, P_7_ immunization induced a significantly higher anti-(S-N fusion)-IgM and anti-(N)-IgM antibody response, compared to its native peptide immunization (p<0.0001; p<0.0001). Also, P_9_ immunization induced a significantly higher anti-(N)-IgM antibody titer, compared to P_10_ (p<0.0001). Membrane-derived lipopeptide and peptide immunizations with P_11_ and P_12_ produced significantly higher anti-(M)-IgM antibody titers, compared to unimmunized controls (p<0.0001) (**Figure 4B III**). Overall, there are systemic IgM antibody responses elicited by intranasal immunizations with individual lipopeptides and peptides against their respective antigens.

Lastly, we measured systemic IgG antibody responses against the S-N fusion, N, and M proteins of SARS-CoV-2. Immunizations with spike-derived and nucleocapsid-derived lipopeptides and peptides induced strong IgG antibody responses to their respective antigens. P_1_-P_4_ immunizations showed significantly higher anti-(S-N fusion)-IgG antibody titres, compared to unimmunized controls (p<0.0001) (**Figure 4B IV**). Anti-(S-N fusion)-IgG antibody titres produced from P_1_ was significantly higher compared to its native peptide immunization (p<0.0001). Notably, P_3_ and P_4_ immunizations produced the highest anti-(S-N fusion)-IgG antibody titer compared to all other immunization groups. All nucleocapsid-derived lipopeptide and peptide immunizations have significantly higher anti-(N)-IgG antibody titres, compared to unimmunized controls (p<0.0001) (**Figure 4B V**). P_5_ and P_9_ immunizations had significantly higher anti-(N)-IgG antibody titres, compared to their respective native peptide immunizations, with p=0.0036 and p=0.0089, respectively. Furthermore, we found that anti-(M)-IgG antibodies were only produced by P_11_ immunizations (**Figure 4B VI**). Other membrane-derived lipopeptides and peptides did not elicit M protein-specific IgG antibody responses. All in all, systemic IgG antibody responses were induced by intranasal immunizations with spike- (P_1_-P_4_), and nucleocapsid- (P_5_-P_10_) derived lipopeptide and peptide immunizations to their respective antigens.

### Intranasal immunizations with spike-derived lipopeptides (P_1_ and P_3_) induce mucosal IgA and systemic IgM/IgG antibody responses reactive across variant spike antigens

**Figure 5:**
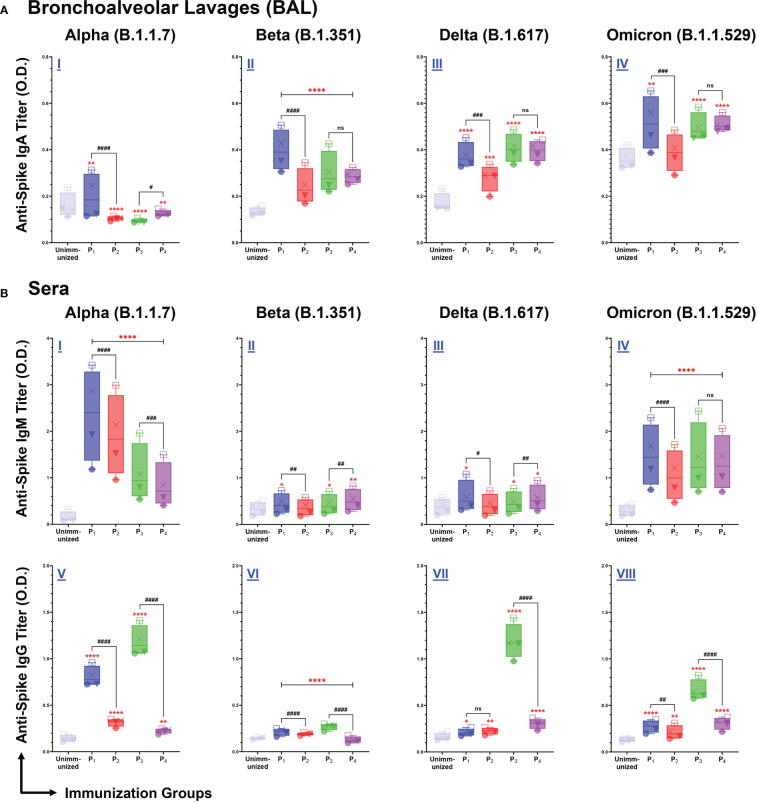
Intranasal immunizations with lipopeptides P_1_ and P_3_ derived from the spike antigens of SARS-CoV-2 induces strong mucosal and systemic antibody responses that are cross-reactive against the Alpha (B.1.1.7), Beta (B.1.351), Delta (B.1.617), and Omicron (B.1.1.529) Variants of SARS-CoV-2. Male C57BL/6 mice (n=3/group) were immunized intranasally with individual lipopeptide (P_1_ or P_3_, 10 μg/mouse), twice, 14 days apart. Eight days after the second immunization, mice were euthanized, and BAL and serum samples were collected. Spike proteins of the Alpha (B.1.1.7), Beta (B.1.351), Delta (B.1.617), and Omicron (B.1.1.529) variants of SARS-CoV-2 were used to coat ELISA plates at 1μg/ml. Plates were incubated with serial dilutions of BALs (□1:2, X1:4, ▼1:8, ●1:16) and sera (□1:100, X1:200, ▼1:400, ●1:800) from immunized mice. The detection of IgA in BAL **(A)**, and IgM/IgG in serum **(B)** are represented by box and whisker plots. Unimmunized mice were used as negative control. Statistical differences were determined by two-way ANONA, followed by Tukey’s test. Comparisons between the immunized and the unimmunized groups are indicated by *p ≤ 0.05, **p ≤ 0.01, ***p ≤ 0.001, and ****p ≤ 0.0001. Comparisons between the lipopeptide and peptide immunized groups are indicated by ns (not significant), ^#^p ≤ 0.05, ^##^p ≤ 0.01, ^###^p ≤ 0.001, and ^####^p ≤ 0.0001. These data points are representative of three independent repeat experiments.

We assessed BAL and serum antibody responses elicited by intranasal immunizations with spike-derived (P_1_-P_4_) lipopeptides and peptides against SARS-CoV-2 VOCs: Alpha (B.1.1.7), Beta (B.1.351), Delta (B.1.617), and Omicron (B.1.1.529). P_1_ immunization showed significantly higher mucosal IgA antibody titers, compared to unimmunized controls and its respective peptide immunization, against all SARS-CoV-2 VOCs (**Figure 5A I-IV**). Additionally, mucosal IgA antibodies induced by P_2_, P_3_, and P_4_ immunizations showed significantly higher titers against B.1.351, B.1.617, and B.1.1.529 variants, compared to unimmunized controls (**Figure 5A II-IV**). These results demonstrated that intranasal immunizations with spike-derived lipopeptides and peptides elicited a lung mucosal IgA antibody response against the B.1.1.7, B.1.351, B.1.617, and B.1.1.529 variants of SARS-CoV-2.

Next, we examined systemic IgM antibody responses against the SARS-CoV-2 VOCs. We found P_1_-P_4_ immunizations induced a significantly higher IgM antibody response, against the B.1.1.7 and B.1.1.529 variants, compared to unimmunized controls (p<0.0001) (**Figure 5B I, IV**). In addition, significantly higher serum IgM antibody titers were elicited by P_1_, P_3_, and P_4_ immunizations against the B.1.351 and B.1.617 variants, compared to the unimmunized controls (**Figure 5B I, II**). P_1_ immunizations have significantly higher IgM responses compared to their respective peptide immunizations, against B.1.1.7 (p<0.0001), B.1.351 (p=0.0049), B.1.617 (p=0.0188), and B.1.1.529 (p<0.0001) variants of SARS-CoV-2 (**Figure 5B I-IV**). Similarly, P_3_ immunizations have significantly higher IgM responses against B.1.1.7, compared to P_4_ (p=0.0001) (**Figure 5B I**). However, the systemic IgM response induced by P_4_ immunizations were significantly higher compared to P_3_ immunizations, against the B.1.351 (p=0.0032) and B.1.617 (p=0.0088) variants (**Figure 5B II, III**). Overall, P_1_-P_4_ immunizations have shown to induce a robust systemic IgM antibody response against all SARS-CoV-2 VOCs.

From serum samples collected from P_1_-P_4_ immunizations, we assessed systemic IgG antibody responses against the four SARS-CoV-2 VOCs. We found all spike-derived lipopeptide and peptide immunizations elicited significantly higher IgG antibody titers compared to unimmunized controls, against all four SARS-CoV-2 VOCs (**Figure 5B V-VIII**). Notably, P_1_ immunizations had significantly higher IgG antibody titers compared to the native peptide immunization, against the B.1.1.7 (p<0.0001), B.1.351 (p<0.0001), and B.1.1.529 (p=0.0059). Similarly, P_3_ immunizations induced significantly higher IgG antibody titers compared to P_4_, against the B.1.1.7, B.1.351, B.1.617, and B.1.1.529 variants (p<0.0001). Evidently, the spike-derived lipopeptide immunizations induced a more robust cross-variant IgG antibody response, compared to their respective peptide immunizations.

### Intranasal immunizations with spike-derived lipopeptides induce higher levels of neutralizing antibodies compared to peptide immunizations, in lungs and systemic blood

**Figure 6:**
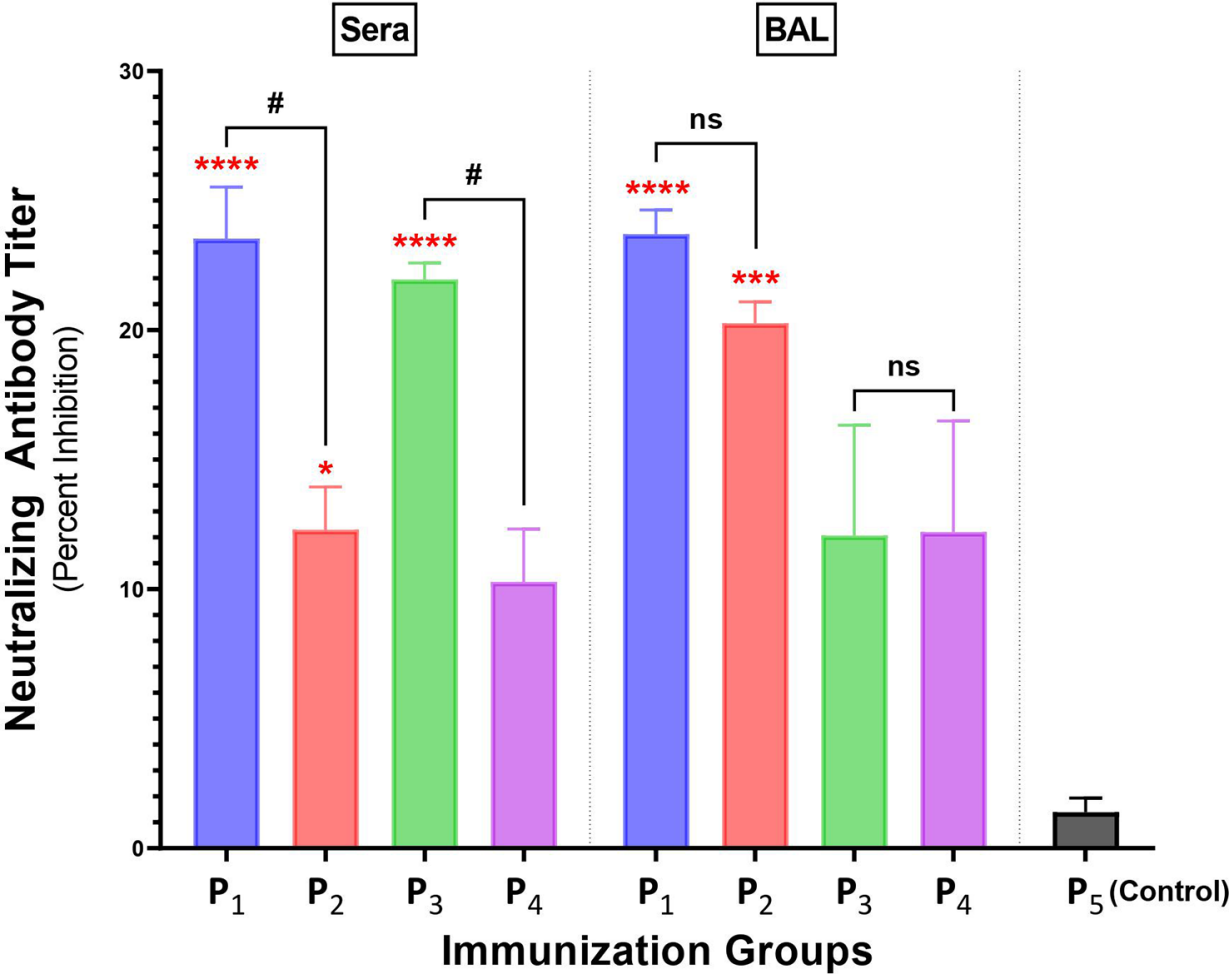
Intranasal immunizations with spike-derived lipopeptides P_1_ and P_3_ induces a strong neutralizing antibody response in lung mucosa and blood. Male C57BL/6 mice (n=3/group) were immunized intranasally with individual lipopeptide (P_1_, P_3_, or P_5_; 10 μg/mouse), twice, 14 days apart. Eight days after the second immunization, mice were euthanized and blood and bronchoalveolar lavages (BALs) were collected. To determine neutralizing antibody responses against SARS-CoV-2, serum (1:9) and BAL (1:1) samples were used in a surrogate viral neutralization assay. The assay was done in triplicates and data is represented as mean ± SEM, from three independent repeat experiments. Serum samples collected from mice immunized with a non-spike lipopeptide (P_5_, derived from N protein) was used as negative control. Statistical significance was determined by one-way ANOVA with Šidák multiple comparison test. Significant differences when comparing between immunization groups and P_5_ (control) are indicated by *p ≤ 0.05, ***p ≤ 0.001, and ****p ≤ 0.0001. Comparisons between lipopeptide and peptide immunizations are indicated by ns (not significant), and ^#^p ≤ 0.05.

Antibody responses in BAL and serum samples collected from P_1_-P_4_ immunizations were tested for their ability to block/neutralize viral binding to target host ACE2 receptor, using a cPass SARS-CoV-2 Neutralization kit. Serum from a non-spike lipopeptide immunization, P_5_, was used as a negative control. Serum antibody responses elicited by P_1_ and P_3_ immunizations showed a percent inhibition of 23.5% and 22.0%, respectively (**Figure 6**), whereas P_2_ and P_4_ immunizations had a percent inhibition of 12.3% and 10.3%, respectively. These results demonstrate that serum antibody responses induced by spike-derived lipopeptides (P_1_ and P_3_) had significantly higher neutralizing capabilities compared to their respective peptides (p=0.0391 and 0.0295, respectively). Next, we found that BAL antibody responses from P_1_ (23.7%) and P_2_ (20.3%) immunization showed significantly higher neutralizing capabilities compared to P_5_ (p<0.0001; p<0.0003), whereas P_3_ (12.1%) and P_4_ (12.2%) immunizations showed similar neutralization capability.

### Phenotypic flow cytometry analysis of immune cells from spleen and lungs of mice immunized with lipopeptides

**Figure 7:**
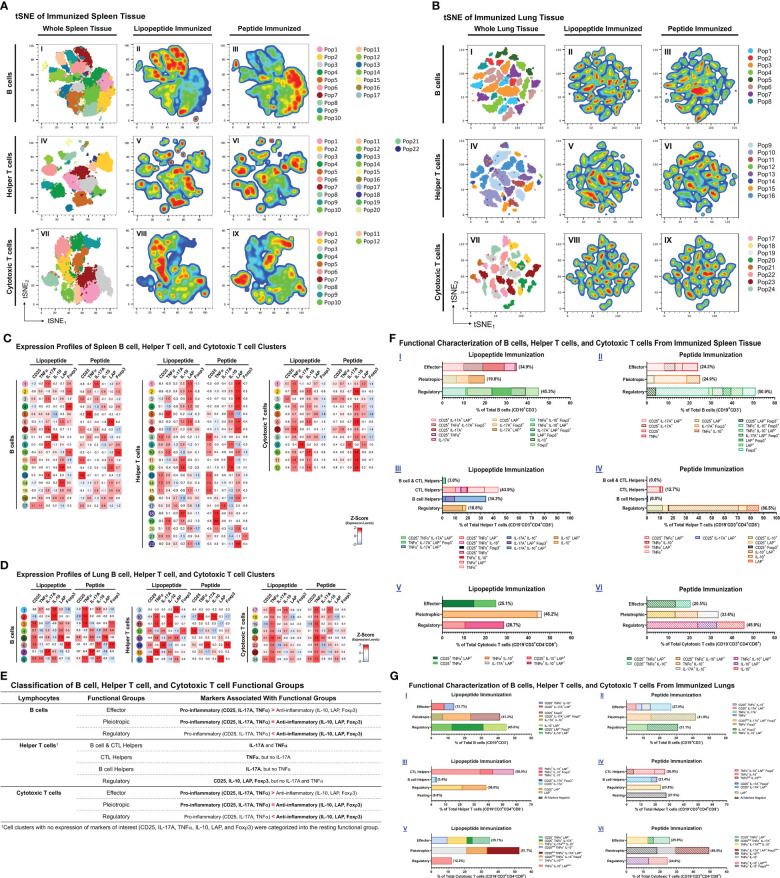
Phenotypic analysis of B cells, helper T cells, and cytotoxic T cells from lungs and spleens of mice immunized with individual lipopeptides and peptides. Flow cytometry data from all individually immunized groups (P_1_- P_14_; n=14 samples) were concatenated, for respective spleen and lung tissues. Concatenated tSNE plots for spleen **(A)** and lung **(B)** tissues were generated, with each cluster indicated by color and Pop#, for B cells (I), Helper T cells (IV), and Cytotoxic T cells (VII). After concatenation, flow data was gated into a combined lipopeptide (P_1_, P_3_, P_5_, P_7_, P_9_, P_11_, and P_13_; n=7 samples) and peptide (P_2_, P_4_, P_6_, P_8_, P_10_, P_12_, and P_14_; n=7 samples) immunized groups to create pseudocolor density plots for B cells (II and III), Helper T cells (V and VI), and Cytotoxic T cells (VIII and IX). In red are cell clusters in high density, and blue represents low density areas. Heatmaps of spleen **(C)** and lung **(D)** tissues show the expression profiles of B cell (left), Helper T cell (middle), and Cytotoxic T cell (right) clusters from the combined lipopeptide and peptide immunized groups. **(E)** Based on the expression profiles of cell clusters and the functional role that the expressed markers play in anti-viral immunity, B cell, Helper T cell, and Cytotoxic T cell clusters were organized into functional groups for the spleen **(F)** and lung **(G)** tissues. The frequency of each cell cluster, assigned to a functional group, is indicated by a colored bar, and its expression profile is outlined in the legend.

Because lipopeptide immunizations induced higher mucosal and systemic antibody titers and splenocyte proliferation responses compared to peptide immunizations, we performed a comprehensive flow cytometric analysis of B cells (CD19^+^CD3^-^), helper T cells (TH, CD19^-^CD3^+^CD4^+^CD8^-^), and cytotoxic T cells (CTL, CD19^-^CD3^+^CD4^-^CD8^+^) to identify functional changes among the immunization groups. Using clustering analysis, we identified cell clusters (Pop#) within B cells, TH and CTLs (**Figures 7A, B**). Next, we characterized cell clusters by examining expression profiles, and compared cell cluster frequencies between the lipopeptide and peptide immunized groups (**Figures 7C, D**). Based on the cell cluster characterizations, we categorized each cell cluster, within B cells, TH cells, and CTLs, into functional groups to determine differences between lipopeptide and peptide immunizations (**Figures 7E–G**).

In the spleen, lipopeptide immunizations demonstrated higher percentages of B cells with effector properties (34.9% vs. 24.2%), TH cells with B cell and CTL helper function (3.0% vs. 0.0%), CTL helper function (43.9% vs. 12.7%), and B cell helper function (34.3% vs. 0.8%), and CTLs with effector (25.1% vs. 20.5%) and pleiotropic (46.2% vs. 33.6%) properties, compared to peptide immunizations (**Figure 7F**). In contrast, peptide immunizations have higher percentages of pleiotropic (24.9% vs. 19.8%) and regulatory (50.9% vs. 45.3%) B cells, regulatory (86.5% vs 18.8%) TH, and regulatory (45.9% vs. 28.7%) CTLs, compared to lipopeptide immunizations.

In the lungs, lipopeptide immunizations induced a lower percentage of effector B cells (13.7% vs. 27.0%), and higher percentage of regulatory B cells (45.0% vs. 31.1%), compared to peptide immunizations (**Figure 7G I, II**). Within the TH clusters, peptide immunizations demonstrated a higher percentage of B cell helper TH cells, compared to lipopeptide immunizations (21.4% vs. 3.4%) (**Figure 7G III, IV**). Lipopeptide immunizations showed higher percentages of CTL helper TH (58.0% vs. 26.9%), and regulatory TH cells (38.6% vs. 23.8%), compared to peptide immunizations. There was a higher percentage of effector CTLs (35.1% vs. 25.9%), and a lower percentage of regulatory CTLs (12.2% vs. 24.6%), in the lipopeptide-immunized compared to the peptide-immunized (**Figure 7G V, VI**).

## Discussion

There is a need to develop a SARS-CoV-2 vaccine, which can induce protective immune responses including both cellular and humoral arms of the immune system, against conserved regions from multiple antigens. Establishing this immunity begins with efficient antigen uptake and processing by APCs, which can lead to downstream T and B cell activation and priming (30). The ability of APCs to efficiently internalize and process vaccine antigens has shown to be associated with factors such as vaccine construct, solubility, and size (30). Hamley et al. have demonstrated that peptides covalently attached to a single palmitoylated lipid chain self-assembles into spherical micelle-like particles (30–32). The lipopeptide constructs investigated in this study have similar capabilities of self-assembly and micelle formation (**Figure 1**). Moreover, studies on macrophage phagocytosis have shown that soluble protein/peptide antigens are inefficient at uptake and cross-presentation compared to particulate/aggregated antigens (33, 34). Therefore, the lipopeptides investigated here may be more readily internalized, processed, and cross-presented on MHC molecules. Furthermore, particulate antigens, ranging from 20 nm-3 μm in diameter, have been shown to have similar efficiencies in cross-presentation by both MHC class I and MHC class II in macrophages (30). In addition to efficient APC uptake and presentation, aggregated antigens can enter lymphatic vessels, drain into lymph nodes, and directly activate T and B cells to induce immune responses. Particles between 20-200 nm efficiently enter the lymphatics, whereas larger particle must be transported *via* specialized cells, such as APCs (30). In sizing experiments, all investigated lipopeptides aggregated within the size range for efficient APC uptake and cross-presentation, however, only P_1_, P_5_, and P_9_ lipopeptides aggregated within the size range for efficient lymphatic vessel entry (**Figure 1**).

The upregulation of antigen-presenting (HLA-DR) and costimulatory (CD40/CD86) molecules demonstrate enhanced capacity to present antigen on APCs and prime T cells. Interestingly, we found that lipopeptides derived from SARS-CoV-2 antigens led to higher upregulation of HLA-DR, CD40, and CD86 in comparison to the corresponding peptides and medium-alone control (**Figures 2A, B**). The co-upregulation of HLA-DR, CD40, and CD86 on APCs by lipopeptides highlights the initiation of T cell activation with signal 1 (TCR interaction with the antigen-MHCII complex) and signal 2 (interaction of costimulatory molecules) requirements being met (35, 36). This allows APCs to activate CD4^+^ TH, and CD8^+^ CTL mechanisms, and establish antigen-specific effector responses. The purpose of vaccination is to establish pathogen-specific, long-lasting, protective immunity which is primarily mediated by the adaptive arms of the immune system. Therefore, the role of APCs becomes critical in initiating adaptive responses against vaccine antigens and bridging the innate and adaptive arms of the immune system (37). Our results suggest that lipopeptides (P_1_, P_3_, P_5_, P_9_, P_11_, and P_13_) activated APCs more efficiently, compared to their respective peptides, enabling the integration of the innate and adaptive systems without the need of an adjuvant. Furthermore, we found that lipopeptides did not stimulate TLR-2 and -4 receptors or produce detectable amounts of proinflammatory cytokines (**Figure 2C**). These results suggest that the designed lipopeptides have the capability of stimulating antigen-presenting function in the absence of TLR-2/4 stimulation and inflammatory responses. This property of the designed lipopeptides is advantageous to avoid any potentially harmful inflammatory side effects of the vaccine. In contrast, SARS-CoV-2 antigens have been shown to activate both TLR2 and TLR4, e.g., S activates both TLRs-2 and -4, and E and N activate TLR-2, leading to strong and potentially harmful inflammatory responses (38). These potent innate-immune stimulation abilities of viral antigens undermine their use as whole antigens in repeated vaccination strategies and support the design and investigation of subunit-based vaccines for repeated use and/or booster doses.

Clonal expansion of antigen-specific T cells by proliferation upon encounter with the relevant peptide-MHC complex is the hallmark of initiating cellular immunity. In gross splenocyte proliferation assays, we found intranasal immunizations with lipopeptides (P_1_, P_3_, P_5_, P_9_, and P_13_) induced significantly higher recall responses, upon *ex vivo* re-stimulation by corresponding lipopeptides and native peptides, compared to peptide immunizations (**Figure 3A**). Importantly, a robust proliferation response against the native peptides upon respective lipopeptide immunizations demonstrates the specificity towards their SARS-CoV-2 S-, N- and M-derived peptide epitopes and not to the lipid tail. However, with P_7_ and P_11_ lipopeptide immunizations, there was no significant antigen-specific proliferation response observed (**Figure 3A IV, VI**). Intriguingly, P_7_ was the only lipopeptide that also did not significantly upregulate HLA-DR, CD40, and CD86. Therefore, P_7_ proliferation responses may be dampened due to inefficient antigen-presentation, inadequate T cell activation by costimulatory molecules, and overall reduced APC activation. Similarly, the proliferation response induced by P_11_ immunizations can be explained by APCs increasing antigen-presentation function by HLA-DR, however the lack of co-stimulatory molecules (CD40/CD86) may drive T cells into anergic states. Anergic conditions are not desirable for a vaccine as it demonstrates the failure of our immune system to mount a response against the targeted antigen (39).

The balance between T_H_1 (IFN-γ, TNF-α, IL-2) and T_H_2 (IL-4) cytokines have been suggested to modulate immune responses towards cell-mediated or humoral immunity. IFN-γ functions to educate immune cells to recognize and eliminate pathogens, and associates with protective immunity against intracellular pathogens (40). Furthermore, TNF-α and IL-2 promote T cell survival and expansion, and maintain long-term effector cell functionality (41, 42). Reports from convalescent COVID-19 patients and vaccinated individuals showed that T_H_1 cytokines (IFN-γ, IL-2) marked the presence of long-lasting, antigen-specific cellular responses (43, 44). Conversely, IL-4 shifts immune responses towards humoral immunity by inducing the differentiation of naïve CD4^+^ T cells into T_H_2 cells, increasing B cells antigen-presenting function, and promoting IgG_1_ secretion (45). However, recent single cell RNA sequencing analyses challenge the T_H_1/T_H_2 dogma and depict a functional continuum, plasticity, and heterogeneity of T cell subsets capable of appropriate immune function under various physiological conditions (46). In our study, cytokine responses from antigen-specific splenocytes demonstrated patterns of increased IFN-γ, TNF-α, IL-2 and IL-4 cytokine production in the lipopeptide immunized groups (**Figure 3B**). Additionally, we found antigen-specific proliferation significantly correlated with only T_H_1 cytokines, suggesting the development of a strong cell-mediated response (**Figure 3C**). Moreover, T_H_1 cytokines can also contribute to humoral responses as IL-2 production has shown to correlate with serum IgG levels, and TNF-α acting as a potential autocrine growth factor for B cell activation and expansion (45, 47). Furthermore, in COVID-19 recovered patients, CD4^+^ T cells producing high levels of IFN-γ and IL-2 displayed polyfunctional phenotypes that stimulated long-lasting memory T and B cell populations (48). Although we did not study memory responses, flow cytometry analysis shows that splenic CD4^+^ T cells, from lipopeptide immunizations, demonstrated the ability to stimulate both humoral (B cell clusters) and cellular (cytotoxic T cell clusters) immune responses (**Figure 7F**). We functionally categorized B cell and CTL clusters into effector, pleiotropic, or regulatory groupings, based on the expression of pro-inflammatory (CD25, IL-17A, TNF-α) and anti-inflammatory (IL-10, LAP, Foxp3) markers (**Figure 7E**) (49–52). The effector group consists of cell clusters associated with immune activation whereas, the regulatory group consists of cell clusters associated with suppression and regulation of immune responses. The pleiotropic functional group represents cell clusters that show a balance between stimulation and suppression/regulation of the immune response. TH cell clusters were organized into B cell and CTL helper, CTL helpers, B cell helpers, and regulatory functional groups. IL-17-producing CD4^+^ T cells are associated with B cell proliferation and antibody isotype class switching responses, therefore TH clusters expressing IL-17, were assigned to the B cell helper functional group (53). Moreover, TNF-α-producing CD4^+^ T cells have shown to stimulate T-cell mediated inflammation and induce protective activity against intracellular pathogens by activating effector CD8^+^ T cells (41, 47, 54). Therefore, TH clusters producing TNF-α were assigned to the CTL helper functional group. On the contrary, the regulatory functional group consists of cell clusters only expressing CD25, IL-10, LAP, or Foxp3 as these markers suppress and regulate CD4^+^ T cell immune responses (55). In the spleens of lipopeptide immunized mice, there were higher percentages of CTL and B cell helper TH populations, which coincided with higher percentages of effector cytotoxic T cell and B cell populations compared to the peptide immunizations. Moreover, the higher percentage of regulatory TH cells coincided with a higher percentage of regulatory CTLs and B cells in the peptide immunized mice. These data revealed that lipopeptide immunizations are activating both cellular and humoral effector response, whereas peptide immunizations induce more regulatory responses in the spleen. CD4^+^ TH cell population displaying poly-functional phenotypes may suggest that the lipopeptide vaccine candidate can establish memory responses. Also, these results highlight the importance of TH populations and their role in orchestrating cellular and humoral immunity. Moreover, a well-coordinated cell-mediated response shows signs of a robust multi-functional and cross-reactive immune response. T cell-mediated responses are highly resistant to mutations acquired by SARS-CoV-2 VOCs, and prevent immune escape of VOCs (11). Furthermore, targeting conserved regions of S, N, and M proteins allows vaccine-induced immunity to cross-react with multiple SARS-CoV-2 variants and broadly cover internal and external components of the virus. This increases the T cell epitope repertoire and potentially further hinders the ability of the virus to escape. Majority of current vaccines and boosters are spike-based and generate variant-specific responses that limit immune coverage of SARS-CoV-2 components, and cross-reactivity towards SARS-CoV-2 variants. Vaccines solely targeting the SARS-CoV-2 spike protein serve well for short-term mitigation, and emergence situation of SARS-CoV-2 pandemic; highlighted by the need for repeated booster shots. To address the long-term issues of continuously emerging SARS-CoV-2 variants and novel coronaviruses, vaccine development approaches need to explore additional avenues in targeting conserved, non-spike proteins as well. Based on these considerations, we will combine the individual lipopeptides studied here into a lipopeptide-mix vaccine candidate in future studies to induce comprehensive coverage against SARS-CoV-2 and its variants.

An effective mucosal vaccine must establish protective immunity at the lung mucosa and prevent SARS-CoV-2 infections at the point of entry. Our initial lipopeptide-based vaccine design was based on the premise that mice immunized intranasally with lipopeptides will induce both mucosal and systemic antibodies which can bind to the respective viral proteins. The lipopeptide immunized groups overall had significantly higher antibody (both mucosal IgA and systemic IgM/IgG) responses, compared to the corresponding peptide groups (**Figure 4**). Furthermore, P_1_ and P_3_ immunizations (both lipopeptides from conserved regions of the spike protein) demonstrated a cross-reactive mucosal IgA and systemic IgM/IgG responses against four different SARS-CoV-2 VOCs (Alpha, Beta, Delta, and Omicron) (**Figure 5**). In addition, neutralizing antibodies can serve to prevent SARS-CoV-2 infections by significantly blocking viral binding to the target host cell receptor, or preventing viral entry, by targeting the RBD or S_2_’ domains, respectively. P_1_ immunizations showed neutralizing antibodies in lung mucosa and systemic blood, whereas P_3_ immunizations led to neutralization antibody responses in the blood (**Figure 6**). Clinically, >15% inhibition in the surrogate viral neutralization assay has been shown to be effective for viral neutralization (56). The induction of high levels of antigen-specific mucosal IgA responses, cross-reactive towards multiple highly variant spike proteins, that also possess neutralization capacity, is a significantly advantageous feature of our vaccine candidate against a highly variable respiratory virus like SARS-CoV-2. The current mRNA vaccines produce an insufficient mucosal response to protect the respiratory tract, the site of infection for SARS-CoV-2, thereby limiting the vaccines’ ability to prevent infections. By inducing cross-variant mucosal IgA, clinically-effective nAb titers in BAL, and poly-functional B/T-lymphocyte profiles in lung tissue, our vaccine candidate shows signature of a robust mucosal response that can potentially lead to a preventative vaccine.

In conclusion, our results clearly demonstrate that intranasal immunization with lipopeptides, even without any added adjuvant, lead to significant induction of mucosal antibody responses and systemic cellular and humoral immune responses. This study describes an important and innovative finding that opens new avenues for developing a broadly protective vaccine for SARS-CoV-2 virus and its variants, and potentially other heterologous coronaviruses.

## Methods

### Synthetic lipopeptides and native peptides

Synthetic lipopeptides (P_1_, P_3_, P_5_, P_7_, P_9_, P_11_, and P_13_) and their corresponding native peptides (P_2_, P_4_, P_6_, P_8_, P_10_, P_12_, and P_14_, respectively), derived from highly conserved and functional regions of the S-, N- and M-proteins of SARS-CoV-2, were custom synthesized by Genscript Inc. (NJ, USA) with >96% purity (**Table 1**). The lipopeptides and peptides were stored at -20**^°^** C in DMSO, at 10 mg/ml, and diluted with PBS or culture medium prior to use.

### Nanoparticle sizing

All lipopeptides were dissolved in PBS at various concentrations, ranging from 0.01 to 1.0 mg/ml. Samples were loaded on a Zetasizer Ultra nanoparticle sizer (Malvern Panalytical, UK), and a standardized Sizing and Zeta report was generated by Zetasizer XPLORER software v1.10 to provide mean particle size for each sample.

### THP-1 monocytes cell culture

THP-1 monocytes (ATCC TIB-202) were grown in medium containing RPMI-1640 (Gibco), 10% fetal bovine serum (FBS), 1% penicillin-streptomycin (P/S), and 1% L-glutamine, and maintained at a confluency between 70-80%. THP-1 cells (2×10^6^ cells/ml) were seeded with P_1_-P_14_, at 1 μg/ml. The cultures were incubated in 5% CO_2_, at 37**^°^** C for 24 hours. The activation of THP-1 monocytes was determined by flow cytometry analysis. A total of 2×10^5^ THP-1 cells from each culture was stained with co-stimulatory markers (anti-human CD86-PE, anti-human CD40-PE, anti-human HLA-DR-APC; Thermo Fisher Scientific, UK) using established procedures from Thermofisher (57). Mean fluorescence intensity (MFI) and percent positive cells (P) values were acquired for each culture. These values were used to calculate _i_MFI values (_i_MFI = MFI × P), and subsequently, performed a percent of control calculation (Percent of Control =100 × _i_MFI_Exp._/_i_MFI_Control_) for graphing (58). Data was expressed as mean ± SEM (standard error mean) of triplicate cultures.

### TLR-2/4 cell cultures

HEK-Blue™ hTLR2 and HEK-Blue™ hTLR4 (Invivogen, CA, USA) cell lines are stably transfected with human TLR2 and TLR4 genes. Upon TLR signalling, a secreted embryonic alkaline phosphatase (SEAP) reporter gene is induced, and SEAP is secreted, which is detected by a QUANTI-Blue™ detection assay.

HEK-Blue™ hTLR2 and HEK-Blue™ hTLR4 cell lines were grown according to their respective handling procedures. HEK-Blue™ hTLR2 (5×10^5^ cells/ml) and HEK-Blue™ hTLR4 (2.5×10^5^ cells/ml) cells were seeded with P_1_-P_14_, at 1 μg/ml and 10 μg/ml, in triplicates. The cultures were incubated in 5% CO_2_, at 37**^°^** C for 24 hours, and supernatants were collected to run QUANTI-Blue™ detection according to the assay protocol. Using a DTX 880 Plate Reader (Beckman Coulter), optical density (OD) readings were taken of QUANTI-Blue™ detection assays, at 620nm. Data was expressed as mean ± SEM of triplicate cultures.

### Mice immunizations

Animal experiments in the study were approved by University of Alberta’s Animal Care and Use Committee (ACUC) for Health Sciences and were conducted according to the guidelines of the Canadian Council of Animal Care (CCAC). Four to six-week-old male C57BL/6 mice were purchased from Charles River Laboratory and housed in a pathogen-free animal facility (HSLAS) at the University of Alberta. Mice were immunized twice, 14 days apart with individual lipopeptides or peptides (10 μg/mouse), intranasally in a total volume of 30 μL (15 μL in each nostril). Mice were euthanized 8 days after second immunization and bronchoalveolar lavages (BAL), sera, lungs, and spleens were collected. Unimmunized mice were used as controls.

### Antigen-specific splenocyte proliferation assay

Eight days after the second immunization, spleens were harvest from euthanized mice and pooled from three mice. Single cell suspensions of pooled spleens, from various immunized groups, were achieved by following a standardized Bio-protocol (59). The splenocytes were resuspended in growth media containing RPMI-1640 (Gibco), 10% FBS, 1% P/S, 1% L-glutamine and 2-mercaptoethanol (2 μL). Proliferative responses were measured in triplicate, in flat-bottom 96-well plates. A total of 4×10^5^ cells splenocytes from immunized mice and 4×10^5^ antigen presenting cells (irradiated splenocytes at 3000 rads, derived from unimmunized mice) were incubated with corresponding lipopeptides and peptides at various concentrations described in the figure legends (60). Plates were incubated for 4 days, and culture supernatants were collected for antigen-specific cytokine analysis, before adding BrdU labelling solution for 18h. A Roche Cell Proliferation ELISA, BrdU colorimetric kit (Sigma-Aldrich, MO, USA) was used to determine BrdU incorporation in proliferating splenocytes, and OD readings were acquired by a DTX 880 Plate Reader (Beckman Coulter, CA, USA), set at 450nm. OD readings were subtracted by APC+Splenocytes background and represented as the mean OD ± SEM of triplicate culture.

### Isolation of lung lymphocytes

Lungs were collected and pooled from three mice, and lung lymphocytes were isolated using a standardized bio-protocol (61). Lung lymphocytes were resuspended in media containing RPMI-1640 (Gibco) with 10% FBS and 1% P/S, for flow cytometry analysis.

### Flow cytometry analysis of lungs and spleens

Splenocytes (2×10^5^ cells) and isolated lung lymphocytes (1×10^5^ cells) from immunized mice were stained with extracellular (anti-mouse CD19-SB600, CD3-NovaFluor Red 710, CD8-NovaFluor Yellow 610, CD4-NovaFluor Red 660, CD25-SB702; Thermo Fisher Scientific, UK) and intracellular markers (anti-mouse TNFα-eFluor 450, IL-10-FITC, LAP-PE, Fox, 3-PE-Cy5, IL-17A-PE-Cy7; Thermo Fisher Scientific, UK) using established protocols from Thermofisher (57, 62). Splenocytes and lung lymphocytes from immunized mice were stimulated with PMA (50 ng/ml) and ionomycin (500 ng/ml) for 20h at 37**^°^** C. Supernatants were collected for cytokine analysis, before adding Brefeldin A (1.5μg/ml) 1X for 4h at 37**^°^** C and subsequently stained with extracellular and intracellular markers as mentioned above. Samples were run on Attune NxT flow cytometer (BD Life Sciences, CA, USA) and analyzed by FlowJo v10.8 Software. Lung and spleen samples from immunized groups P_1_-P_14_ were concatenated to provide consistencies in clustering methods and creating tSNE (t-distributed stochastic neighbor embedding) plots. Using Phenograph and FlowSOM clustering algorithms, lung lymphocytes and splenocytes were classified into different clusters based on phenotypic markers that were expressed, and overlayed on tSNE plots. Violin Box was used to acquire MFI values of phenotypic markers that each cluster expressed, which was represented by heatmaps, created on Morpheus (63).

### Cytokine analysis

Cytokine concentrations were measured from supernatants collected from antigen-specific splenocyte proliferation cultures of P_1_-P_14_ immunized groups by Meso Scale Discovery U-PLEX immunoassays (Meso Scale Diagnostics, MD, USA). The U-PLEX Biomarker Group 1 (mouse) assay was used to analyze cytokines profiles of IFNγ, IL-10, IL-2, IL-4, and TNFα. The MSD plates were read on MESO QuickPlex SQ120 and analyzed on DISCOVERY WORKBENCH 4.0 Analysis Software.

### Antigen specific antibody ELISA

Serum and BAL samples were pooled from three immunized mice and ran in duplicates for each dilution, on 96-well plates. The plates were coated with SARS-CoV-2 nucleocapsid, membrane, a spike-nucleocapsid fusion protein, and the spike proteins of Alpha (B.1.1.7), Beta (B.1.351), Delta (B.1.617), and Omicron (B.1.1.529) variants (GenScript lnc., NJ, USA). For detection of IgG and IgM in serum, and IgA in BAL, the antibody ELISA procedure described earlier was followed (64). The absorbance was read using DTX 880 Plate Reader (Beckman Coulter), and data was represented as box plots.

### Antibody neutralization assay

Serum and BAL samples from P_1_-P_4_ immunized groups were tested for neutralizing antibodies. Triplicate samples were run using the cPass SARS-CoV-2 Neutralization Antibody Detection Kit according to the manufacturer’s instructions. Serum and BAL samples were run at 1:9 dilutions and undiluted, respectively, and read using a DTX 880 Plate Reader (Beckman Coulter). Data are expressed as mean ± SEM of triplicates.

### Graphs and statistical analysis

Data were analyzed, and graphed using GraphPad Prism Software 9.4.1(CA, USA). Data was presented as mean ± SEM of 2-3 replicate values of 3 pooled mice and statistical differences were analyzed by one-way or two-way ANOVAs, adjusted for multiple comparisons. In addition, Spearman’s test was performed to determine the correlations between cytokines and proliferation values. A *P* ≤ 0.05 was used to indicated significance.

## Data availability statement

The raw data supporting the conclusions of this article will be made available by the authors, without undue reservation.

## Ethics statement

The animal study was reviewed and approved by University of Alberta’s Animal Care and Use Committee (ACUC) for Health Sciences (AUP00000212) and according to the guidelines of the Canadian Council of Animal Care (CCAC).

## Author contributions

RP, design, planning, execution of experiments, data analyses, writing first draft and editing. BA, conception, funding, design, planning, supervision, data analyses, writing and editing. All authors contributed to the article and approved the submitted version.
